# Supplementary material for: The causal relationship between the human gut microbiota and pyogenic arthritis: a Mendelian randomization study
Source: Front Cell Infect Microbiol. 2024 Nov 26;14:1452480. doi: 10.3389/fcimb.2024.1452480 (PMC11629706; doi:10.3389/fcimb.2024.1452480)
Supplement: Supplementary file 1 [file DataSheet1.zip › mendelian test/ebi-a-GCST90027781.csv_leave_one_out.pdf]

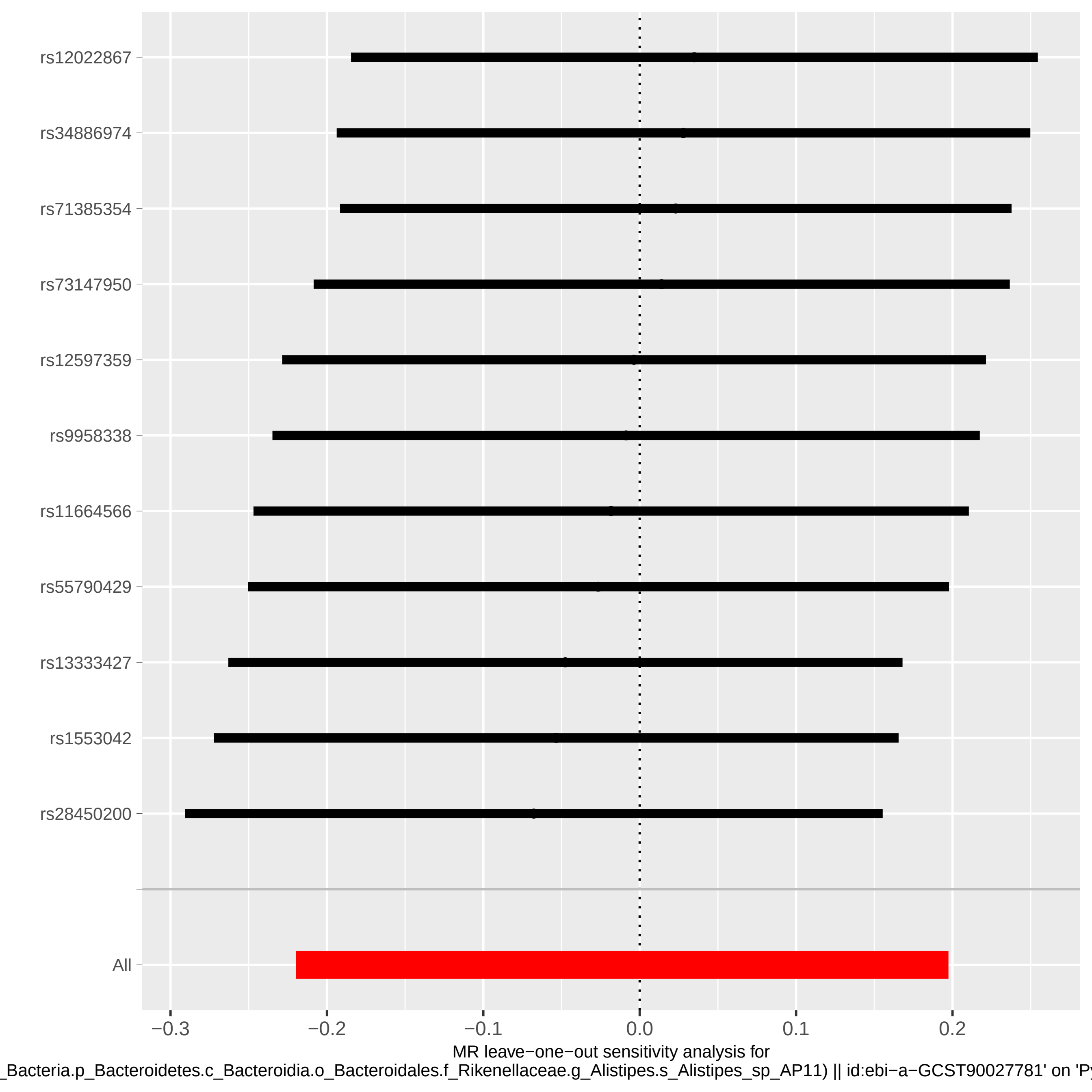

rs12022867
rs34886974
rs71385354
rs73147950
rs12597359
rs9958338
rs11664566
rs55790429
rs13333427
rs1553042
rs28450200
All
−0.3
−0.2
−0.1
0.0
0.1
0.2
MR leave−one−out sensitivity analysis for
_Bacteria.p_Bacteroidetes.c_Bacteroidia.o_Bacteroidales.f_Rikenellaceae.g_Alistipes.s_Alistipes_sp_AP11) || id:ebi−a−GCST90027781' on 'P
